# Kidney function trajectories, associated factors, and outcomes in multiethnic Asian patients with type 2 diabetes

**DOI:** 10.1111/1753-0407.13523

**Published:** 2024-01-02

**Authors:** Liang Feng, Yong Mong Bee, Xiuju Fu, Jia Liang Kwek, Choong Meng Chan, Tazeen H. Jafar

**Affiliations:** ^1^ Program in Health Services & Systems Research Duke‐NUS Medical School Singapore Singapore; ^2^ Department of Endocrinology Singapore General Hospital Singapore Singapore; ^3^ Institute of High Performance Computing A*STAR Singapore Singapore; ^4^ Department of Renal Medicine Singapore General Hospital Singapore Singapore; ^5^ Duke Global Health Institute Durham North Carolina USA

**Keywords:** albuminuria, estimated glomerular filtration rate, latent class linear mixed models, risk factors, trajectories, type 2 diabetes

## Abstract

**Background:**

We examined the trajectory of estimated glomerular filtrate rate (eGFR), associated risk factors, and its relationship with end‐stage kidney disease (ESKD) among a multiethnic patient population with type 2 diabetes in Singapore.

**Methods:**

A follow‐up study included 62 080 individuals with type 2 diabetes aged ≥18 years in a multi‐institutional SingHealth Diabetes Registry between 2013 and 2019. eGFR trajectories were analyzed using latent class linear mixed models. Factors associated with eGFR trajectories were evaluated using multinomial logistic regression. The association of eGFR trajectories with ESKD was assessed via competing risk models.

**Results:**

Trajectory of kidney function, determined by eGFR, was nonlinear. The trajectory pattern was classified as stable initially then gradual decline (75%), progressive decline (21.9%), and rapid decline (3.1%). Younger age, female sex, Malay ethnicity, lower‐income housing type, current smoking, higher glycated hemoglobin, lower low‐density lipoprotein, higher triglyceride, uncontrolled blood pressure, albuminuria, cardiovascular disease, hypertension, and higher eGFR levels each were associated with progressive or rapid decline. Compared with the trajectory of stable initially then gradual eGFR decline, progressive decline increased the hazard of ESKD by 6.14‐fold (95% confidence interval [CI]: 4.96–7.61)) and rapid decline by 82.55 folds (95% CI: 55.90–121.89).

**Conclusions:**

Three nonlinear trajectory classes of kidney function were identified among multiethnic individuals with type 2 diabetes in Singapore. About one in four individuals had a progressive or rapid decline in eGFR. Our results suggest that eGFR trajectories are correlated with multiple social and modifiable risk factors and inform the risk of ESKD.

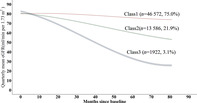

## Graphical abstract

- Three estimated glomerular filtration rate (eGFR) trajectories were identified among Asian individuals with type 2 diabetes: stable initially then gradual decline (75%), progressive decline (21.9%), and rapid decline (3.1%)
- Multiple factors were associated with progressive or rapid eGFR decline (eg, women, low socioeconomic status, high hemoglobin A_1c_, and albuminuria)
- Progressive and rapid eGFR decline were associated with a higher risk of end‐stage kidney disease (ESKD)
- Our results highlight the gender and social disparities in renal function decline and also underscore the potential benefit of multifactorial intervention targeting the modifiable risk factors to postpone ESKD.

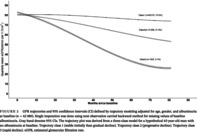

## 1 INTRODUCTION

The global prevalence of diabetes mellitus (DM) has increased greatly in the past few decades^1^ and has been projected to increase by about 17% (237 million) between 2019 and 2045,^2^ which will lead to a substantial growth of diabetic kidney disease (DKD) and associated end‐stage kidney disease (ESKD). Asians are known to be more susceptible to diabetes than Western populations,^3^ and much of the increase in DM has been in Asia.^1^ In many Asian countries such as Singapore, South Korea, and Japan, >40% of ESKD is attributable to DM.^4^ However, the chronic trajectory of kidney function among patients with diabetes is not well established.

Considerable variations in the patterns or trajectories of estimated glomerular filtration rate (eGFR) have been reported among patients with diabetes.^5^, ^6^, ^7^, ^8^, ^9^, ^10^, ^11^ The heterogeneity in kidney function trajectory in patients with diabetes highlights the need for risk stratification of those at risk of accelerated loss of renal function, early institution of preventive therapy, and close monitoring. However, very few studies have been conducted among Asians with diabetes on longitudinal kidney function trajectories.^9^, ^11^, ^12^ Also, previous studies were restricted to patients with preserved baseline renal function^9^ or included mostly males.^12^

We used trajectory modeling to describe trajectories of kidney function in patients with type 2 diabetes based on data from SingHealth Diabetes Registry (SDR),^13^ a multi‐institutional diabetes registry in Singapore, from 2013 to 2019. We aimed to (a) characterize the different trajectories of eGFR changes over time before the occurrence of ESKD, (b) identify risk factors associated with various patterns of eGFR decline, and (c) define the relationship of the trajectories to ESKD.

## 2 METHODS

### 2.1 Population

Our data on individuals with type 2 diabetes were selected from SDR.^13^ The SDR was established in 2015 and has been populated with data retrospectively and prospectively to cover the period from 2013 to 2019. It is updated annually, with patient data from electronic medical records across the primary and hospital‐based care continuum within SingHealth‐the largest healthcare cluster in Singapore. SingHealth comprises four acute hospitals, five national specialty centers, three community hospitals, and eight primary care clinics (SingHealth Polyclinics),^14^ which cumulatively provide health services to ~50% of the population of Singapore. Individuals with diabetes were identified through diagnosis codes (International Classification of Diseases, Ninth Revision [ICD‐9], ICD‐10, Systematized Nomenclature of Medicine [SNOMED], and SingHealth Polyclinic Working Diagnosis Code [SHWKC]), prescription records for diabetes medications, or laboratory tests (fasting plasma glucose, oral glucose tolerance test, hemoglobin A_1c_ [HbA_1c_]).^13^ The SDR includes data on patient demographics, prescribed and dispensed medications, comorbidities, anthropometrics, laboratory tests, and health services utilization.

Using data from SDR, we identified 94 145 individuals with type 2 diabetes aged ≥18 years in 2013 (baseline of the study) and 81 329 of them had eGFR measurements at baseline. The study was approved by the National University of Singapore Institutional Review Board and SingHealth Centralised Institutional Review Board. Informed consent was not sought because the study analyzed anonymized datasets from the SDR in a sandbox environment. No human or animal studies involved or no ethical statement for the study.

### 2.2 Measurement

#### 2.2.1 Study outcomes

The primary study outcome was the chronic eGFR trajectories evaluated by quarterly average eGFR. GFR was calculated using the Chronic Kidney Disease Epidemiology Collaboration equation based on levels of serum creatinine, age at the time of serum creatinine measurement, race, and gender.^15^ The secondary outcome was ESKD. Data on ESKD between 2013 and 2019 were traced in the Singapore Renal Disease Registry.

#### 2.2.2 Covariates

The study baseline was 2013. Covariates included baseline age (18 to 65 years and ≥ 65 years), gender, ethnicity (Chinese, Indian, Malay, and others), socioeconomic status (SES), body mass index (BMI), smoking, HbA_1c_ (<7.0%, ≥7 and ≤9%, and >9.0%), blood pressure (BP) control status, low‐density lipoprotein cholesterol (LDL‐C), high‐density lipoprotein cholesterol (HDL‐C), triglyceride, albuminuria, eGFR, cardiovascular disease (CVD), hypertension, and use of lipid‐lowering medication. SES was evaluated by housing type (one‐ to two‐room House Development Board [HDB] flats, three‐ to five‐room HDB flats, and condo or landed houses). BMI was calculated as weight in kilograms divided by the square of height in meters. Smoking was defined as current smoking (yes/no). Uncontrolled BP was defined as systolic BP ≥ 140 mm Hg or diastolic BP ≥ 90 mm Hg. Albuminuria was defined as either urine albumin and creatinine ratio (UACR) ≥30 mg/g or positive results for microalbuminuria by Micral strip test (Roche Diagnostics, Singapore). CVD was defined as the presence of coronary heart disease, heart failure, or stroke, which were identified by ICD‐10, SNOMED, and SHWKC. Hypertension was defined as the use of any antihypertensive drugs.

#### 2.2.3 Statistical analysis

Continuous variables were described using mean ± SD or median and interquartile range (IQR), as appropriate. We compared means (or medians) and proportions between eGFR trajectory groups using analysis of variance (Kruskal–Wallis test, if appropriate), or the *χ* ^2^ test.

#### 2.2.4 eGFR trajectories

eGFR trajectories were analyzed by modeling quarterly average eGFR using latent class linear mixed models.^16^ We considered for each individual the eGFR measurements until ESKD, death, or the end of 2019. The number of quarters since baseline was considered as time of this data point in modeling and was converted to months when creating figures. Polynomial terms for follow‐up time, including linear, quadratic, and cubic terms, were specified to model the nonlinear trajectories. The random intercept term was specified in the model to account for individual departures from the mean trajectory of each class. At model convergence, each individual was assigned posterior likelihoods of belonging to each eGFR trajectory. Individuals were assigned to the class to which they had the highest likelihood of belonging. eGFR trajectories were evaluated in models controlling for baseline age, gender, and albuminuria. Missing data on baseline albuminuria were replaced by the next available follow‐up observation for the same individual. Information on albuminuria was still missing on 13 965 individuals after single imputation. We fitted the models with one to five classes and selected the optimal number by considering Bayesian information criterion (BIC), Akaike information criterion (AIC), posterior probability of membership (an average posterior probability >0.85 for all latent classes), sample size of each class (no less than 1% participants in any single trajectory class), and interpretability of patterns.^17^, ^18^ BIC and AIC values to the models adjusted for age, gender, and baseline albuminuria decreased with the increasing number of hypothesized classes of respective models and no models had a class of <1% of individuals (Table S1). Thus, neither BIC nor AIC nor sample size of an individual class was informative for the model selection. Of all the five models, only models with two and three classes had a mean posterior probability of >85% for all classes of the same model. We finally selected the model with three classes instead of two classes because it is more clinically interpretable.

As a comparison, we also applied traditional linear mixed‐effects regression to quarterly average eGFR and obtained the slope, assuming a linear decline in eGFR. Sensitivity analysis was also done by constructing unadjusted trajectory models and adjusted trajectory models where all missing baseline albuminuria was singly imputed via predictive mean matching (PMM) method.

#### 2.2.5 Factors associated with eGFR trajectories

Factors associated with eGFR trajectories were evaluated using multinomial logistic regression. The baseline covariates were chosen based on previous studies^11^, ^19^ including age, gender, ethnicity, housing type, smoking, Hb1Ac, LDL‐C, HDL‐C, triglyceride, albuminuria, eGFR, BP control, CVD, hypertension, and lipid‐lowering medication use. Two models were constructed. Model 1 included age, gender, ethnicity, and housing type, and model 2 was a fully adjusted model including all aforementioned covariates. Because the majority of BMI data at baseline were missing, sensitivity analysis was done by adding baseline BMI into model 2. Available baseline BMI (*n* = 23 540) or baseline BMI imputed using the next available follow‐up value of the same individual (*n* = 60 541) was controlled for, respectively.

#### 2.2.6 Association of eGFR trajectories with ESKD

Death before occurrence of ESKD and ESKD were considered as competing risks. Competing risk analysis was thus performed using Fine and Gray models^20^ to evaluate the risk of ESKD associated with eGFR trajectories. Covariates in the models were the same as those in the fully adjusted logistic regression model. Due to missing data on covariates (Table 1), we conducted sensitivity analysis by repeating multinomial logistic regression and competing risk analysis based on multiply‐imputed data. We used multivariate imputation by chained equations to create 10 imputed datasets with the predictive mean matching method. Variables used in each imputation model were all the covariates plus trajectory class and ESKD except for those with a minimum proportion of usable cases of <25%. All imputations were done through the R package “mice.”^21^

**TABLE 1:** Baseline (year 2013) characteristics and incident events of individuals with type 2 diabetes by eGFR trajectory groups (*n* = 62 080).

Variables	Total (*n* = 62 080)	Class 1 (*n* = 46 572)	Class 2 (*n* = 13 586)	Class 3 (*n* = 1922)	*p* value
Baseline characteristics					
Age at entry (years), mean (SD)	64.1 (11.4)	64.2 (11.6)	64.5 (10.3)	60.4 (11.0)	<.001
18 to 65, *n* (%)	31 509 (50.8)	23 566 (50.6)	6711 (49.4)	1232 (64.0)	<.001
≥65, *n* (%)	30 571 (49.2)	23 006 (49.4)	6875 (50.6)	690 (35.9)	
Female	31 644 (51.0)	22 999 (49.4)	7549 (55.6)	1096 (57.0)	<.001
Housing type, *n* (%)					
1 to 2 rooms HDB	4663 (7.9)	3328 (7.5)	1135 (8.7)	200 (11.0)	<.001
3 to 5 rooms HDB	48 254 (81.2)	36 086 (80.9)	10 675 (82.2)	1493 (82.4)	
Condo or landed house	6478 (10.9)	5179 (11.6)	1180 (9.1)	119 (6.6)	
Missing, *n*	2685	1979	596	110	
Ethnicity, *n* (%)					<.001
Chinese	44 702 (72.0)	33 985 (73.0)	9536 (70.1)	1181 (61.5)	
Indian	6311 (10.2)	4924 (10.6)	1190 (8.8)	197 (10.3)	
Malay	8558 (13.8)	5823 (12.5)	2299 (16.9)	436 (22.7)	
Others	2509 (4.0)	1840 (4.0)	561 (4.1)	108 (5.6)	
Body mass index (kg/m^2^)					
Mean (SD)	26.4 (4.5)	26.3 (4.5)	26.7 (4.6)	26.5 (4.9)	<.001
Missing, *n*	38 540	29 071	8301	1168	
HbA_1c_ (%)					
Median (IQR)	7.1 (6.5, 7.8)	7.0 (6.5, 7.7)	7.2 (6.6, 8.0)	7.8 (6.8,9.8)	<.001
<7.0%, *n* (%)	27 373 (44.8)	21 580 (47.1)	5292 (39.5)	501 (26.6)	<.001
≥7 and ≤ 9%, *n* (%)	27 254 (44.6)	20 110 (43.9)	6377 (47.6)	767 (40.7)	
>9.0%, *n* (%)	6466 (10.6)	4123 (9.0)	1725 (12.9)	618 (32.8)	
Missing, *n*	987	759	192	116	
Systolic blood pressure (mm Hg)					
Mean (SD)	131.0 (12.9)	130.2 (12.6)	133.0 (13.1)	135.7 (15.5)	<.001
Missing, *n*	3742	2728	724	290	
Diastolic blood pressure (mm Hg)					
Mean (SD)	70.0 (8.0)	69.9 (7.9)	70.3 (8.0)	72.2 (9.0)	<.001
Missing, *n*	3742	2728	724	290	
Uncontrolled BP^a^, *n* (%)	12 654 (21.7)	8703 (19.9)	3395 (26.4)	556 (34.1)	<.001
Missing, *n*	3742	2728	724	290	
HDL cholesterol (mg/dl)					
Mean (SD)	51.6 (13.7)	51.7 (13.8)	51.3 (13.6)	49.4 (13.4)	<.001
Missing, *n*	2305	1687	490	128	
LDL cholesterol (mg/dl)					
Mean (SD)	91.7 (27.5)	91.8 (27.1)	90.4 (27.6)	97.8 (33.2)	<.001
Missing, *n*	2647	1885	591	171	
Triglyceride (mg/dl)					
Median (IQR)	115.1 (88.6, 159.4)	115.1 (88.6, 159.4)	124.0 (88.6, 168.3)	132.4 (97.4, 186)	<.001
Ln (triglyceride), mean (SD)	4.8 (0.45)	4.8 (0.44)	4.8 (0.45)	4.9 (0.49)	<.001
Missing, *n*	2247	1637	483	127	
Albuminuria^b^, *n* (%)	34 173 (55.1)	24 349 (52.3)	8459 (62.3)	1365 (71.0)	<.001
eGFR (ml/min/1.73 m^2^), mean (SD)	82.4 (22.5)	82.4 (23.2)	81.5 (20.3)	87.7 (18.6)	<.001
CVD, *n* (%)	14 349 (23.1)	10 517 (22.6)	3319 (24.4)	513 (26.7)	<.001
Hypertension^c^, *n* (%)	53 734 (86.6)	39 550 (84.9)	12 464 (91.7)	1720 (89.5)	<.001
ACEI or ARB, *n* (%)	43 573 (70.2)	31 549 (67.4)	10 537 (77.6)	1487 (77.4)	<.001
Oral antidiabetic drugs, *n* (%)	50 204 (80.9)	36 613 (78.6)	11 775 (86.7)	1816 (94.5)	<.001
Insulin use, *n* (%)	5265 (8.5)	3535 (7.6)	1319 (9.7)	411 (21.4)	<.001
Lipid‐lowering medication use, *n* (%)	52 317 (84.3)	39 065 (83.9)	11 657 (85.8)	1595 (83.0)	<.001
Incident events					
ESKD, *n* (%)	885 (1.4)	270 (0.6)	316 (2.3)	299 (15.6)	<.001
CVD^d^, *n* (%)	7199 (15.1)	4912 (13.6)	1856 (18.1)	431 (30.6)	<.001
CVD mortality, *n* (%)	1926 (3.1)	1388 (3.0)	428 (3.2)	110 (5.7)	<.001
All‐cause mortality, *n* (%)	5733 (9.2)	4403 (9.5)	1022 (7.5)	308 (16.0)	<.001

Abbreviations: ACEI, angiotensin‐converting enzyme inhibitor; ARB, angiotensin‐receptor blocker; BP, blood pressure; CVD, cardiovascular disease; eGFR, estimated glomerular filtration rate; ESKD, end‐stage kidney disease; HbA_1c_: hemoglobin A_1c_; HDB, Housing Development Board; HDL, high‐density lipoprotein; IQR, interquartile range; LDL, low‐density lipoprotein.

^a^ Systolic BP > 140 mm Hg or diastolic BP > 90 mm Hg.

^b^ Urine albumin creatinine ratio >30 mg/g or abnormal dipstick test, missing values of baseline albuminuria (*n* = 17 004) were imputed using next observation carried backward.

^c^ Defined as use of any antihypertensive medication.

^d^ Patients with CVD at baseline were excluded, and the denominator for the total samples and classes 1 to 3 were 47 731, 36 055, 10 267, and 1409, respectively.

Trajectory analyses was performed using ‘LCMM’ packages in R 4.1.0. Multinomial logistic regression and competing risk analysis were done using SAS, version 9.4 (SAS Institute, Inc, Cary, NC). A *p* value of <.05 was considered to indicate statistical significance.

## 3 RESULTS

### 3.1 Study population

**FIGURE 1:**
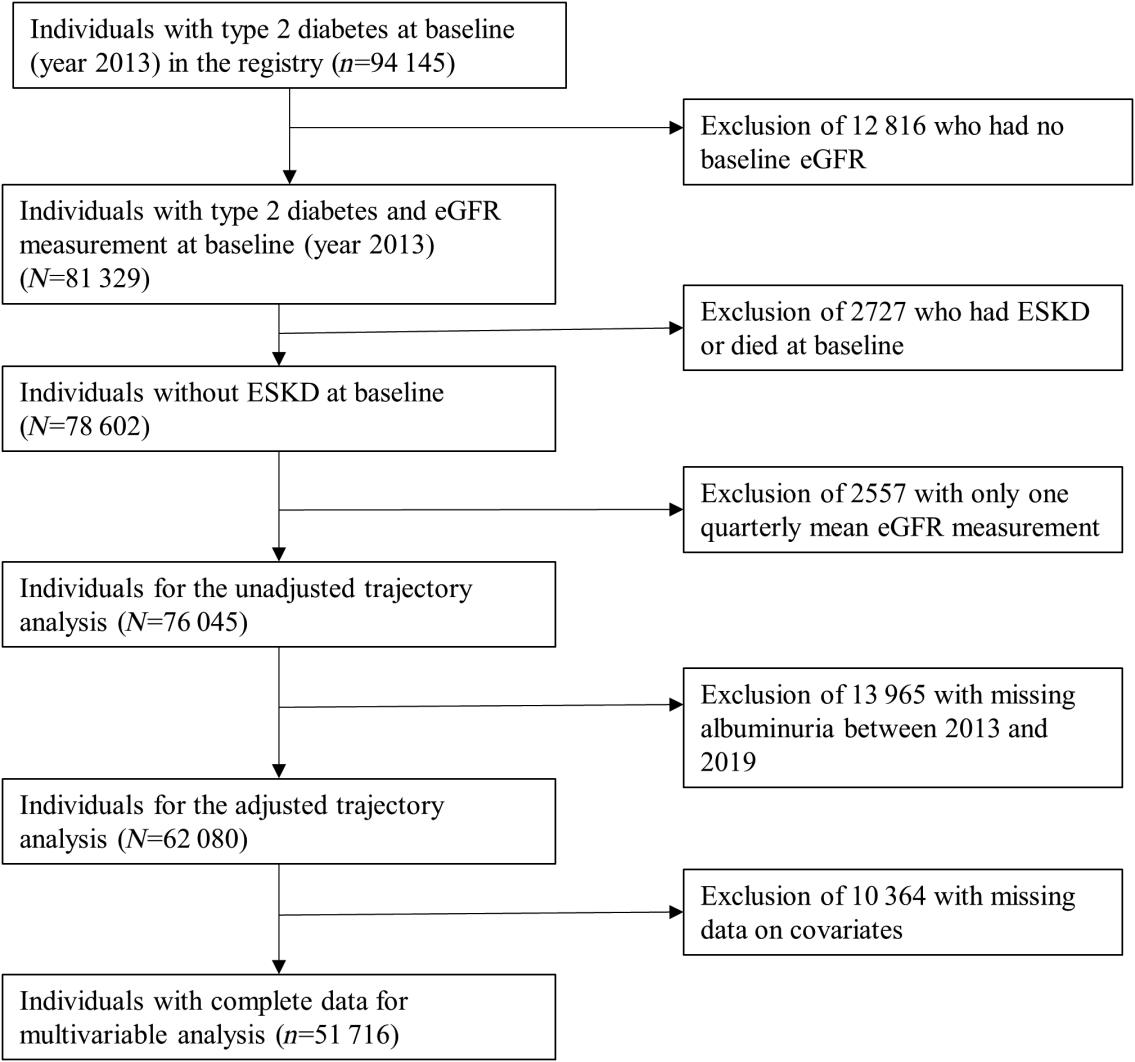
Flow chart showing the selection and follow‐up of study participants. eGFR, estimated glomerular filtration rate; ESKD, end‐stage kidney disease.

A total of 81 329 individuals with type 2 diabetes in the SDR had eGFR at baseline. Of them, we excluded those who had ESKD or died at baseline (*n* = 2727), had only one average eGFR measurement between 2013 and 2019 (*n* = 2557) or had no baseline albuminuria (*n* = 13 965); thus final sample size was 62 080 individuals for adjusted trajectory analysis (Figure 1) The mean (SD) age of the 62 080 individuals was 64 (11.4) years, about half (51.0%) were women, 72% were Chinese, and 55% had albuminuria (30 mg/g or higher) (Table 1). Table S2 compares the characteristics of the 62 080 individuals with those excluded from the adjusted trajectory analysis (*n* = 13 965).

Of the cohort of 62 080 individuals with diabetes, 885 (1.4%) had incident ESKD during a median follow‐up period of 72 months (IQR: 69–78). The incident rate of ESKD was 2.24 (95% CI: 2.10–2.40) per 1000 person‐years during follow‐up.

There was a median of 11 (IQR: 8–19) serum creatinine (or eGFR) measurements per individual, and 1 045 769 measurements in total. This number was reduced to 9 (IQR:7–12) eGFR per individual and altogether 633 208 measurements after merging serial eGFR within a quarter (3 months) into a mean value, which was used in the trajectory modeling.

### 3.2 Trajectory of eGFR

**FIGURE 2:**
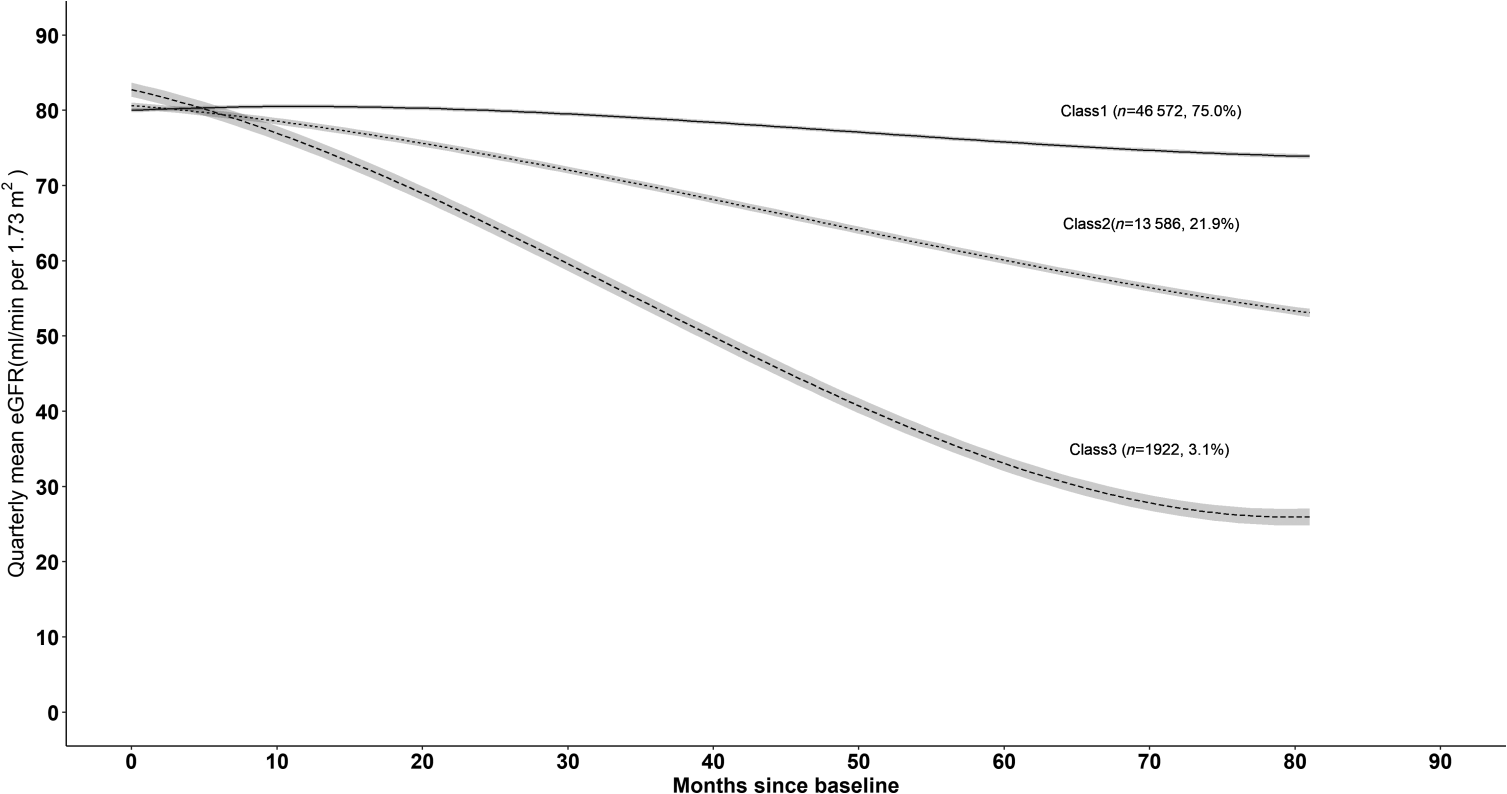
GFR trajectories and 95% confidence intervals (CI) defined by trajectory modeling adjusted for age, gender, and albuminuria at baseline (*n* = 62 080). Single imputation was done using next observation carried backward method for missing values of baseline albuminuria. Gray band denotes 95% CIs. The trajectory plot was derived from a three‐class model for a hypothetical 65‐year‐old man with no albuminuria at baseline. Trajectory class 1 (stable initially then gradual decline). Trajectory class 2 (progressive decline). Trajectory class 3 (rapid decline). eGFR, estimated glomerular filtration rate.

Figure 2 represents the trajectories with three classes for a hypothetical man who is 65 years old and has no albuminuria at baseline. Similar trajectories were observed for a woman aged 65 years with no albuminuria at baseline (Figure S1). We named trajectory classes from adjusted models as follows: class 1, stable initially then gradual decline; class 2, progressive decline; and class 3 rapid decline. The majority of the individuals were in class 1 (*n* = 46 572, 75.0%), followed by those in class 2 (*n* = 13 586, 21.9%) and in class 3 (*n* = 1922,3.1%). Parameter estimates are reported for the adjusted three‐class model in Tables S3 to S5. Also, a plot of estimated mean trajectory in combination with the observed individual trajectories of 100 randomly selected individuals from each class indicated that individual variability was generally explained by latent group trajectories (Figure S2). According to the results from traditional linear mixed model, the rates of eGFR decline with adjustment for age were −1.06 (−1.07, −1.05), −4.54 (−4.57, 4.52), and − 9.93 (−9.99, 9.86) mL/min/1.73 m^2^ per year for individuals in classes 1 to 3, respectively (Table 2).

**TABLE 2:** Age‐adjusted annual change in eGFR in different trajectories.

	% of sample	Mean (SD) baseline eGFR (mL/min/1.73 m^2^)	Slope (95% CI) (mL/min/1.73 m^2^/year)	*p* value
Class 1	76.0	82.4 (23.2)	−1.06 (−1.07, −1.05)	<.001
Class 2	20.6	81.5 (20.3)	−4.54 (−4.57, −4.52)	<.001
Class 3	3.4	87.7 (18.6)	−9.93 (−9.99, −9.86)	<.001

Abbreviations: eGFR, estimated glomerular filtration rate; 95% CI, 95% confidence interval.

### 3.3 Baseline characteristics and incident events by adjusted eGFR trajectories

The baseline characteristics by adjusted eGFR trajectories are shown in Table 1. A higher proportion of individuals with unfavorable cardiovascular profiles were found in the rapid decline (class 3) and progressive decline (class 2) groups compared with those in the group with initially stable eGFR followed by gradual decline (class 1). Similarly, higher proportions incident events were observed in class 2 and class 3 as compared with class 1.

### 3.4 Association of baseline clinical variables with kidney function trajectories

Table 3 summarized the association of baseline factors with eGFR trajectories from multinomial logistic regression with trajectory class 1 (stable initially then gradual decline) being the reference group. In model 1, female (vs male) sex, Malay ethnicity (vs Chinese), and lower‐cost housing type each were associated with increased odds of being in class 2 or 3. With adjustment for additional covariates in model 2, the associations remained significant. In model 2, female sex, Malay ethnicity, lower‐cost housing type, current smoking, higher Hb1Ac, higher triglyceride, albuminuria, higher eGFR levels, uncontrolled BP, CVD, and hypertension each were associated with a higher likelihood of being in both class 2 and class 3 as compared with class 1. For most factors, the association was stronger with class 3 than with class 2. Moreover, age ≥65 years was associated with a lower chance of belonging to class 3, with no impact on class 2. Indian ethnicity compared with Chinese ethnicity and higher LDL‐C levels were associated with a decreased chance of belonging to class 2, without affecting class 3. In contrast, no significant association was identified for HDL‐C and use of lipid‐lowering medication.

**TABLE 3:** Factors of being in class 2 or 3 versus class 1 by multinomial logistic regression.

Variables	Model 1 (*n* = 59 395)				Model 2 (*n* = 57 176)			
	Class 2		Class 3		Class 2		Class 3	
	Adjusted OR (95% CI)	*p*	Adjusted OR (95% CI)	*p*	Adjusted OR (95% CI)	*p*	Adjusted OR (95% CI)	*p*
Age ≥ 65 years (vs 18 to 65 years)	1.05 (1.01, 1.09)	.025	0.60 (0.55, 0.67)	<.001	1.04 (0.99, 1.09)	.13	0.87 (0.76, 0.99)	.045
Gender, women	1.26 (1.21,1.31)	<.001	1.38 (1.25,1.52)	<.001	1.31 (1.25, 1.37)	<.001	1.53 (1.35, 1.73)	<.001
Ethnicity								
Chinese	1.00		1.00		1.00		1.00	
Indian	0.85 (0.80, 0.91)	<.001	1.05 (0.90, 1.23)	.52	0.85 (0.78, 0.91)	<.001	0.88 (0.73, 1.06)	.18
Malay	1.36 (1.28,1.43)	<.001	1.83 (1.62, 2.06)	<.001	1.35 (1.27,1.44)	<.001	1.62 (1.40, 1.87)	<.001
Others	1.08 (0.98, 1.19)	.13	1.61 (1.31, 1.98)	<.001	1.07 (0.96, 1.20)	.23	1.46 (1.14, 1.87)	.003
Housing type								
Condo or landed house	1.00		1.00		1.00		1.00	
1 to 2 rooms HDB	1.41 (1.29,1.56)	<.001	2.20 (1.74,2.78)	<.001	1.26 (1.14,1.40)	<.001	1.40 (1.08,1.83)	.012
3 to 5 rooms HDB	1.26 (1.17, 1.34)	<.001	1.56 (1.29, 1.88)	<.001	1.18 (1.10, 1.27)	<.001	1.22 (0.99, 1.50)	.062
Current smoking (vs not)					1.08 (1.00,1.18)	.065	1.60 (1.33, 1.92)	<.001
HbA1C								
<7.0%					1.00		1.00	
≥7 and ≤ 9%					1.28 (1.22, 1.34)	<.001	1.43 (1.25,1.63)	<.001
>9.0%					1.81 (1.67, 1.95)	<.001	4.41 (3.76, 5.18)	<.001
LDL cholesterol, per 10 mg/dL increase					0.97 (0.96, 0.98)	<.001	1.00 (0.98, 1.02)	.63
HDL cholesterol, per 10 mg/dL increase					0.99 (0.97, 1.01)	.25	0.97 (0.92, 1.02)	.20
Ln (triglyceride cholesterol)					1.18 (1.12, 1.25)	<.001	1.26 (1.09, 1.46)	.002
Albuminuria^a^					1.37 (1.31, 1.43)	<.001	2.17 (1.92, 2.45)	<.001
Baseline eGFR, per 10 mL/min/1.73 m^2^ increase					1.03 (1.02, 1.04)	<.001	1.15 (1.12,1.18)	<.001
Uncontrolled BP^b^					1.33 (1.26, 1.40)	<.001	1.72 (1.52, 1.94)	<.001
Cardiovascular disease					1.07 (1.02, 1.13)	.009	1.38 (1.21, 1.57)	<.001
Hypertension^c^					1.77 (1.64, 1.92)	<.001	1.91 (1.56, 2.33)	<.001
Lipid‐lowering medication					1.02 (0.96, 1.09)	.46	0.90 (0.78, 1.05)	.18

*Note*: Trajectory classes were based on covariate‐adjusted trajectory analysis controlling for baseline age, gender, and albuminuria. Missing values of baseline albuminuria were imputed using next observation carried backward method. Class 1 was the reference group. All variables were baseline measurements in 2013. Because of missing data on covariates 51 716 out of 62 080 were retained for analysis in model 2.

Abbreviations: 95% CI, 95% confidence interval; BP, blood pressure; eGFR, estimated glomerular filtration rate; HbA_1c_: hemoglobin A_1c_; HDB, Housing and Development Board; HDL, high‐density lipoprotein; LDL, low‐density lipoprotein; OR, odds ratio.

^a^ Urine albumin creatinine ratio >30 mg/g or abnormal dipstick test, missing values of baseline albuminuria (*n* = 17 004) were imputed using next observation carried backward method.

^b^ Systolic BP > 140 mm Hg or diastolic BP > 90 mm Hg.

^c^ Defined as use of any antihypertensive.

### 3.5 Association of trajectories with ESKD

Table 4 shows the association between eGFR trajectory and the risk of ESKD from competing risk analysis. Proportional hazard assumption was examined via plots of Schoenfeld residuals versus time, and no violation was found. Relative to class 1 in the unadjusted model, class 2 raised the hazard of ESKD by 4.07 times (95% CI: 3.46–4.79), whereas class 3 increased it by 29.15 times (95% CI: 24.72–34.38). The associations were even stronger after controlling for confounding factors (hazard ratio [HR]: 6.14, 95% CI: 4.96–7.61 for class 2; HR: 82.55, 95% CI: 55.90–121.89 for class 3). After baseline eGFR was excluded from the model, the resulting adjusted HRs became markedly smaller (class 2: HR: 3.22, 95% CI: 2.65–3.92; Class 3: HR: 15.92, 95% CI: 12.66–20.03).

**TABLE 4:** Association of eGFR trajectory group with ESKD from 2013 to 2019.

Class	No. of events	Events per 1000 person‐year	Unadjusted		Adjusted^a^	
			HR (95% CI)	*p* value	HR (95% CI)	*p* value
1	270	0.91	1.00		1.00	
2	316	3.6	4.07 (3.46, 4.79)	<.001	6.14 (4.96,7.61)	<.001
3	299	26.5	29.15 (24.72, 34.38)	<.001	82.55 (55.90, 121.89)	<.001

*Note*: 51 716 out of 62 080 were retained for adjusted analysis because of missing data on covariates.

Abbreviations: eGFR, estimated glomerular filtration rate; ESKD, end‐stage kidney disease; HR, hazard ratio; 95% CI, 95% confidence interval.

^a^ Adjusted for baseline characteristics including age, gender, ethnicity, housing type, smoking, hemoglobin A1c, low‐density lipoprotein cholesterol, high‐density lipoprotein cholesterol, triglyceride, blood pressure control status, albuminuria, cardiovascular disease, hypertension (ie, any antihypertensive drugs use), lipid‐lowering medication use, and eGFR.

### 3.6 Sensitivity analysis

We further conducted unadjusted trajectory analysis and adjusted trajectory analysis with missing baseline albuminuria imputed by PMM method. A similar pattern of change and class size were identified from both unadjusted (Figure S3) and adjusted models (Figure S4), suggesting the robustness of our trajectory analysis results.

Also, additional adjustment of baseline BMI or analysis of multiply imputed data yielded comparable results on factors associated with eGFR trajectories (Tables S6 and S7). The association of eGFR trajectories with ESKD was not materially changed in the analysis of multiply imputed data (Table S8).

## 4 DISCUSSION

Our analysis of longitudinal eGFR trajectories on 62 080 patients with type 2 diabetes before the onset of ESKD identified three distinct nonlinear trajectory patterns showing that eGFR in the majority remained stable initially then declined gradually (class 1), or one in five patients showed a progressive decline (class 2), and a small minority had a rapid decline (class 3) in eGFR. We observed that female sex, Malay ethnicity, lower‐cost housing type, current smoking, higher Hb1Ac, higher triglyceride, albuminuria, higher eGFR levels, uncontrolled BP, CVD, and hypertension each were positively associated with both progressive and rapid decline in eGFR. Notably, age ≥ 65 years was exclusively related to a reduced chance of rapid eGFR decline, whereas Indian ethnicity and higher LDL‐C were solely associated with a decreased chance of progressive decline. Progressively and rapidly declining eGFR trajectories were both associated with ESKD. To our knowledge, this is the largest study of its kind examining eGFR trajectories in patients with type 2 diabetes. Understanding trajectories of kidney function before the onset of ESKD may help risk stratification of the patients for close monitoring. Sharing the trajectories can facilitate provider‐patient discussions on the prognosis, and shared decision making about therapies. Additionally, our results highlight the gender and social disparities in renal function decline and also underscore the potential benefit of multifactorial intervention targeting the modifiable risk factors to postpone ESKD.

Our findings of three distinct change patterns of trajectory classes (stable then gradual decline, progressive decline, and rapid decline) are consistent with an earlier, albeit much smaller, study of 6330 Chinese individuals with type 2 diabetes and preserved baseline renal function (eGFR>60 mL/min/1.73 m^2^).^9^ By contrast, another study of 24 723 Chinese participants with prediabetes or diabetes showed that most (about 86%) of the participants had either stable or increasing eGFR trajectories.^12^ A few explanations are possible for the observed differences. First, it has been shown that prediabetes does not predict eGFR decline in the general population.^22^ Second, study participants were mostly men (84%) whose kidney function was reported to be less negatively affected by hyperglycemia than women.^23^ Further data are required to elucidate the longitudinal eGFR trajectories among Asians with diabetes and prediabetes.

We observed that older age (age ≥ 65 years) was associated with a lower risk of rapid eGFR decline. This can be explained by the fact that older patients may pass away due to other diseases before experiencing a rapid eGFR decline. Our results concur with earlier studies showing a higher likelihood of progressive or rapid decline in kidney function among women vs men^24^, ^25^ and patients with low SES,^26^, ^27^ the latter defined as those living in low‐income housing type. The association of women with faster eGFR decline is somewhat controversial. However, estrogen loss could play an important role in explaining the gender disparity observed. Kidney function has been reported to decrease faster in postmenopausal women as compared with men of similar age.^28^ Moreover, women with diabetes seem to lose the protective effects of estrogen on the cardiovascular bed, even before menopause.^24^ Additionally, the unfavorable CVD risk profiles in women with type 2 diabetes might also contribute to their higher risk of kidney function decline.^29^ We have previously shown that living in smaller public housing is associated with an increased risk of type 2 diabetes, and that residing in smaller public housing is likely to be a useful surrogate for lower SES in Singapore.^30^ We now show that living in small public houses is also associated with a faster decline in kidney function. Our findings have implications that although the underlying social determinants of kidney disease in patients with type 2 diabetes need to be addressed, more health care resources for better access to health care could be provided in the areas with more public housing to eliminate the health disparities in the risk of ESKD.

In our study, high HbA_1c_ (>9.0%) was the strongest correlate of progressive and rapid kidney function decline. Higher level of HbA_1c_ has been consistently shown to be a risk factor for rapid kidney function decline among patients with diabetes.^9^, ^11^, ^19^, ^31^ The relationship has been suggested to be possibly mediated by hyperfiltration^31^ and a higher risk of microvascular complications including retinopathy and neuropathy.^32^, ^33^ Lowering HbA_1c_ slows DKD progression.^32^, ^33^, ^34^, ^35^

Higher levels of LDL‐C were associated with a reduced likelihood of progressive eGFR decline in the study. Conflicting results have been reported on their association.^9^, ^36^, ^37^ The reasons for the seemingly counterintuitive relationship are unclear. Of note, low LDL has been associated with high mortality by others probably reflecting comorbid states including malnutrition and inflammation.^38^ It is also possible that patients with lower LDL‐C levels were more likely to use statins and had more risk factors for progression. Although the association persisted with adjustment of relevant confounders, residual confounding or unmeasured confounders that could not be adjusted for may also contribute to our findings. On the other hand, our study identified no significant relationship between LDL‐C and a rapid eGFR decline as previously reported.^39^ Notably, both positive^37^ and negative associations^9^ have been documented in other research, indicating the need for additional data to clarify the relationship between LDL‐C and various patterns of eGFR decline.

Albuminuria is a known predictor of faster eGFR decline^9^, ^40^ and CVD mortality.^41^ Several therapies including renin‐angiotensin‐aldosterone system (RAAS) blockers and sodium‐glucose cotransporter‐2 inhibitors are effective in slowing DKD progression and recommended by the recent 2020 Kidney Disease Improving Global Outcomes guidelines for management of DKD.^42^ The association between elevated baseline eGFR and subsequent steeper eGFR decline was reported in other studies.^8^, ^37^ It probably reflects some patients with hyperfiltration and others who are malnourished from a concurrent illness, both of whom are more likely to have progressive kidney function decline.^43^, ^44^ Concurrent illness maybe associated with decreased muscle mass which could overestimate true GFR.

We found that both hypertension and uncontrolled BP were associated with a progressive and rapid decline in kidney function. Several epidemiological studies demonstrate a bidirectional relationship of hypertension with kidney disease^45^, ^46^, ^47^ and hypertension is an established risk factor for ESKD.^48^, ^49^ Uncontrolled hypertension accelerates the loss of nephrons and hence GFR.^50^ Sodium and water expansion, upregulated RAAS, sympathetic overactivity, endothelial damage, and other cellular mechanisms are some mediators of CKD‐induced hypertension.^50^ Several trials including the UK Prospective Diabetes Study show the benefit of lowering elevated BP in patients with diabetes in slowing progression to ESKD.^51^, ^52^ This study revealed that classes 2 and 3 individuals had greater RAAS blockers usage than class 1, indicating more severe disease and appropriate treatment for the two classes. This underscores the role of renoprotective agents in influencing eGFR trajectory differences. Current smoking and higher triglycerides are potentially modifiable risk factors for faster decline in kidney function in our study and are known predictors of CVD as well.^53^

As noted in earlier studies,^54^, ^55^ we found that both progressive and rapid eGFR decline were associated with a higher risk of ESKD. The decline in kidney function may act as a biological biomarker of adverse vascular conditions^56^ and might also cause decreased appetite and reduced physical function, thereby indirectly contributing to higher ESKD risk.^57^ Furthermore, adjusting for covariates notably strengthened the correlation between eGFR trajectories and ESKD, indicating the presence of potential negative confounders. This negative confounding effect primarily stems from baseline eGFR. It is well established that GFR has a negative association with ESKD,^58^ and our study revealed a positive association between baseline eGFR and subsequent eGFR decline. Moreover, removing baseline eGFR from the multivariable model lead to HRs for ESKD associated with eGFR trajectories smaller than unadjusted HRs, further supporting the role of baseline eGFR as a potential negative confounder. Our data highlight the necessity of earlier intervention based on GFR trajectories to delay the detrimental decline in kidney function and the progression to adverse clinical outcomes such as ESKD.

The main strengths of this study are the largest sample size of multiethnic Asian patients with type 2 diabetes examining eGFR trajectories, a large number of eGFR measurements for most patients over a 7‐year follow‐up, complete follow‐up data on ESKD from a comprehensive diabetes registry, and consistent results on sensitivity analysis. There are several limitations in this study. First, informed presence bias with more frequent analysis of the sicker population leading to potentially spurious associations is always a possibility in the analysis of electronic health record data.^59^ However, the majority of patients with data in SDR visited polyclinics proactively on regularly scheduled appointments and thereby minimizing selection bias. Second, we fitted the model by using mean eGFR value of each follow‐up quarter rather than using all available measurements. Changes in eGFR within a very short period (ie, within a quarter) would be neglected and potentially introduce bias. However, our primary interest was in subacute or chronic changes in eGFR over a longer duration and not short‐term variations, which are more likely to be transient and results of hydration status, side effects of medication, or acute systemic illness. Third, residual confounding cannot be eliminated. Due to a significant proportion of missing data on UACR (92%), we define albuminuria based on UACR and dipstick tests, the latter without being a semiquantitative test, even though small changes in urine albumin can carry distinct risks for ESKD.^60^ However, this approach has been used in previous studies.^61^, ^62^ Also, duration of diabetes was not accounted for in our analysis. Finally, some characteristics of the excluded individuals were different from the included. The excluded, compared with the included, had higher systolic BP, LDL‐C, triglyceride levels, lower eGFR, and a higher proportion of CVD, indicating a worse prognosis. But the analysis results based on data from multiple imputations were consistent. Thus, we believe our findings are robust.

In conclusion, our study revealed three distinct eGFR trajectories of patients with type 2 diabetes in Singapore: (a) stable initially then gradual decline (class 1), (b) progressive decline (class 2), and (c) rapid decline (class 3). Younger age, female sex, Malay (vs Chinese) ethnicity, lower‐cost housing type, current smoking, higher HbA_1c_, lower LDL‐C, higher triglyceride, albuminuria, higher eGFR levels, uncontrolled BP, CVD, and hypertension, each, were associated with a progressive or rapid decline in kidney function. Both progressive decline and rapid decline vs stable initially then gradual decline were associated with a greater risk of ESKD. More studies are needed to explore strategies to reduce the risk of ESKD among patients with diabetes in class 2 and 3.

## CONFLICT OF INTEREST STATEMENT

All the authors declared no competing interests.

## Supporting information


**Data S1.** Supporting Information.
