# Supplementary material for: Kidney function trajectories, associated factors, and outcomes in multiethnic Asian patients with type 2 diabetes
Source: J Diabetes. 2024 Jan 2;16(9):e13523. doi: 10.1111/1753-0407.13523 (PMC11418407; doi:10.1111/1753-0407.13523)

Table S1. Results of the covariate-adjusted † model fitting process

| No. of latent classes | Log likelihood | BIC | AIC | % Participants per class | Mean posterior probability |
| --- | --- | --- | --- | --- | --- |
| 1 | -2359474 | 4719048 | 4718967 | — |  |
| 2 | -2317401 | 4634956 | 4634829 | 13.1/86.9 | 0.912/0.972 |
| 3 | -2306439 | 4613087 | 4612915 | 3.1/21.9/75.0 | 0.920/0.852/0.916 |
| 4 | -2300212 | 4600689 | 4600472 | 2.2/7.8/14.8/75.2 | 0.924/0.810/0.860/0.900 |
| 5 | -2293799 | 4587918 | 4587656 | 1.7/7.1/8.6/9.1/73.6 | 0.922/0.824/0.812/0.840/0.895 |

† Adjusted for age, gender and albuminuria at baseline. Missing albuminuria was imputed via next observation carried backward method

Table S2 baseline characteristics for individuals included and excluded from trajectory analysis.

| Variables | Included  (n=62080) | Excluded (n=13965) | P value |
| --- | --- | --- | --- |
| Age at entry (year), mean (SD) | 64.1 (11.4) | 68.9 (13.2) | <0.001 |
| Women, n (%) | 31644 (51.0) | 6812 (48.8) | <0.001 |
| Housing type, n (%) |  |  |  |
| 1 to 2 rooms HDB | 4663 (7.9) | 1404 (10.5) | <0.001 |
| 3 to 5 rooms HDB | 48254 (81.2) | 10675 (79.9) |  |
| Condo or landed house | 6478 (10.9) | 1276 (9.6) |  |
| Ethnicity, n (%) |  |  | <0.001 |
| Chinese | 44702 (72.0) | 9438 (67.6) |  |
| Indian | 6311 (10.2) | 1488 (10.7) |  |
| Malay | 8558 (13.8) | 2345 (16.8) |  |
| Others | 2509 (4.0) | 694 (5.0) |  |
| HbA_1C_ (%), median (IQR) | 7.1 (6.5, 7.8) | 6.9 (6.2, 8.0) | <0.001 |
|  |  |  |  |
| Systolic BP (mmHg), mean (SD) | 131.0 (12.9) | 137.2 (18.2) | <0.001 |
|  |  |  |  |
| Diastolic BP (mmHg), mean (SD) | 70.0 (8.0) | 69.6 (9.6) | <0.001 |
|  |  |  |  |
| HDL cholesterol(mg/dl), mean (SD) | 51.6 (13.7) | 47.1 (13.5) | <0.001 |
|  |  |  |  |
| LDL cholesterol(mg/dl), mean (SD) | 91.7 (7.5) | 95.8 (32.8) | <0.001 |
|  |  |  |  |
| Triglyceride (mg/dl), |  |  |  |
| Median (IQR) | 115.1 (88.6, 159.4) | 132.9 (97.4, 186.0) | <0.001 |
| Ln (Triglyceride), mean (SD) | 4.8 (0.42) | 4.9 (0.49) | <0.001 |
| eGFR (ml/min/1.73m2), mean (SD) | 82.4 (22.5) | 58.9 (31.0) | <0.001 |
| CVD, n(%) | 14349 (23.1) | 7244 (51.9) | <0.001 |
| Hypertension †, n (%) | 53734 (86.6) | 12305 (88.1) | <0.001 |
| ACEI or ARB, n (%) | 43573 (70.2) | 9069 (64.9) | <0.001 |
| Oral antidiabetic drugs, n(%) | 50204 (80.9) | 9952 (71.3) | <0.001 |
| Insulin use, n (%) | 5265 (8.5) | 2597 (18.6) | <0.001 |
| Lipid-lowering medication use , n(%) | 52317 (84.3) | 10900 (78.1) | <0.001 |

Abbreviations: SD, standard deviation; HDB, Housing Development Board; HbA_1c_ :haemoglobin A_1c_ ; IQR, interquartile range; BP, blood pressure; HDL, high-density lipoprotein; LDL, low-density lipoprotein; eGFR, estimated glomerular filtration rate ; CVD, cardiovascular disease; ACEI, angiotensin-converting enzyme inhibitor; ARB, angiotensin-receptor blocker

† defined as use of any antihypertensive medication

Table S3. Parameter estimates for adjusted† 3-class latent class linear mixed model: fixed effect in the class-membership model

| Polynomial term | Class | Coefficient | SE | Wald | P value |
| --- | --- | --- | --- | --- | --- |
| Intercept | 1 | 1.05 | 0.02 | 58.70 | <0.001 |
| Intercept | 3 | -2.02 | 0.03 | -70.64 | <0.001 |

Note: class 2 is the reference class.

† Adjusted for age, gender and albuminuria at baseline. Missing albuminuria was imputed via next observation carried backward method

Table S4. Parameter estimates for adjusted† 3-class latent class linear mixed model: fixed effect in longitudinal model

| Polynomial term | Class | Coefficient | SE | Wald | P value |
| --- | --- | --- | --- | --- | --- |
| Intercept | 1 | 153.45 | 0.43 | 356.25 | <0.001 |
| Intercept | 2 | 134.72 | 0.48 | 280.15 | <0.001 |
| Intercept | 3 | 106.10 | 0.64 | 165.88 | <0.001 |
| Month | 1 | -2.67 | 0.09 | -29.32 | <0.001 |
| Month | 2 | -9.87 | 0.17 | -57.04 | <0.001 |
| Month | 3 | -9.12 | 0.48 | -19.13 | <0.001 |
| Month^2^ | 1 | 1.79 | 0.10 | 18.21 | <0.001 |
| Month^2^ | 2 | 2.78 | 0.19 | 14.54 | <0.001 |
| Month^2^ | 3 | 15.94 | 0.53 | 30.26 | <0.001 |
| Month^3^ | 1 | 0.81 | 0.03 | 28.47 | <0.001 |
| Month^3^ | 2 | 1.07 | 0.06 | 19.21 | <0.001 |
| Month^3^ | 3 | 4.20 | 0.15 | 27.73 | <0.001 |
| Age | — | -1.13 | 0.01 | -176.64 | <0.001 |
| Male | — | -5.22 | 0.14 | -36.39 | <0.001 |
| Albuminuria | — | -3.22 | 0.15 | -21.99 | <0.001 |

† Adjusted for age, gender and albuminuria at baseline. Missing albuminuria was imputed via next observation carried backward method

Table S5. Parameter estimates for adjusted† 3-class latent class linear mixed model: Variance-covariance matrix of the random-effects

|  | Intercept |
| --- | --- |
| Intercept | 302.55 |

† Adjusted for age, gender and albuminuria at baseline. Missing albuminuria was imputed via next observation carried backward method

Table S6. Association of baseline characteristics with covariate-adjusted † estimated glomerular filtration rate (eGFR) trajectories with baseline body mass index (BMI) as a covariate in the model

| Variables | Available BMI used (n=21153) | | | | |  | Imputed BMI used‡ (n=51009) | | | |
| --- | --- | --- | --- | --- | --- | --- | --- | --- | --- | --- |
|  |  | Class 2 | | Class 3 | |  | Class 2 |  | Class 3 |  |
|  |  | Adjusted OR  (95% CI) | P value | Adjusted OR  (95% CI) | P value |  | Adjusted OR  (95% CI) | P value | Adjusted OR  (95% CI) | P value |
| BMI, kg/m^2^ |  | 1.00 (0.99,1.01) | 0.82 | 0.97 (0.95,0.99) | 0.001 |  | 1.00 (0.99, 1.00) | 0.62 | 0.97 (0.96, 0.99) | <0.001 |
| Age≥65 y (vs 18 to 65 y) |  | 1.05 (0.97, 1.13) | 0.24 | 0.76 (0.62, 0.92) | 0.005 |  | 1.04 (0.99, 1.10) | 0.11 | 0.82 (0.72, 0.94) | 0.005 |
| Gender, women |  | 1.36 (1.26,1.46) | <0.001 | 1.35 (1.12,1.62) | 0.001 |  | 1.32 (1.26, 1.38) | <0.001 | 1.54 (1.36, 1.75) | <0.001 |
| Ethnicity |  |  |  |  |  |  |  |  |  |  |
| Chinese |  | 1.00 |  | 1.00 |  |  | 1.00 |  | 1.00 |  |
| Indian |  | 0.84 (0.75, 0.95) | 0.004 | 0.91 (0.69, 1.20) | 0.50 |  | 0.85 (0.79, 0.92) | <0.001 | 0.93 (0.77, 1.12) | 0.43 |
| Malay |  | 1.39 (1.26,1.53) | <0.001 | 1.65 (1.34, 2.05) | <0.001 |  | 1.36 (1.27,1.44) | <0.001 | 1.71 (1.47, 1.98) | <0.001 |
| Others |  | 0.99 (0.82, 1.18) | 0.88 | 1.68 (1.16, 2.43) | 0.006 |  | 1.08 (0.97, 1.21) | 0.18 | 1.49 (1.15, 1.93) | 0.002 |
| Housing type |  |  |  |  |  |  |  |  |  |  |
| Condo or landed house |  | 1.00 |  | 1.00 |  |  | 1.00 |  | 1.00 |  |
| 1 to 2 rooms HDB |  | 1.26 (1.07,1.48) | 0.005 | 1.58 (1.06,2.37) | 0.025 |  | 1.26 (1.14,1.40) | <0.001 | 1.37 (1.05,1.79) | 0.020 |
| 3 to 5 rooms HDB |  | 1.19 (1.07, 1.33) | 0.002 | 1.43 (1.05, 1.96) | 0.025 |  | 1.19 (1.10, 1.27) | <0.001 | 1.21 (0.98, 1.49) | 0.079 |
| Current smoking (vs. not) |  | 1.11 (0.98,1.26) | 0.096 | 1.32 (1.01,1.73) | 0.045 |  | 1.09 (1.00,1.19) | 0.043 | 1.57 (1.31, 1.90) | <0.001 |
| HbA1C |  |  |  |  |  |  |  |  |  |  |
| <7.0% |  | 1.00 |  | 1.00 |  |  | 1.00 |  | 1.00 |  |
| ≥7 and ≤9% |  | 1.25 (1.16, 1.34) | <0.001 | 1.16 (0.95, 1.41) | 0.15 |  | 1.28 (1.22, 1.34) | <0.001 | 1.43 (1.26,1.64) | <0.001 |
| >9.0% |  | 1.76 (1.57, 1.97) | <0.001 | 4.20 (3.36, 5.25) | <0.001 |  | 1.82 (1.68, 1.96) | <0.001 | 4.52 (3.85, 5.31) | <0.001 |
| LDL cholesterol, per 10 mg/dl increase |  | 0.97 (0.96, 0.98) | <0.001 | 1.00 (0.97, 1.03) | 0.86 |  | 0.97 (0.96, 0.98) | <0.001 | 1.00 (0.98, 1.02) | 0.63 |
| HDL cholesterol, per 10 mg/dl increase |  | 0.98 (0.95, 1.01) | 0.21 | 0.93 (0.86, 1.00) | 0.038 |  | 0.99 (0.97, 1.01) | 0.18 | 0.96 (0.91, 1.01) | 0.11 |
| Ln (Triglyceride cholesterol) |  | 1.20 (1.09,1.31) | <0.001 | 1.13 (0.91,1.39) | 0.26 |  | 1.18 (1.11, 1.25) | <0.001 | 1.31 (1.13, 1.51) | <0.001 |
| Uncontrolled BP§ |  | 1.39 (1.29,1.50) | <0.001 | 1.94 (1.64, 2.30) | <0.001 |  | 1.33 (1.26, 1.40) | <0.001 | 1.75 (1.55, 1.97) | <0.001 |
| Albuminuria ¶ |  | 1.43 (1.34,1.53) | <0.001 | 2.32 (1.95, 2.77) | <0.001 |  | 1.38 (1.32, 1.44) | <0.001 | 2.16 (1.91, 2.44) | <0.001 |
| Baseline eGFR, per 10 ml/min/1.73 m^2^ increase |  | 1.04 (1.02, 1.05) | <0.001 | 1.16 (1.11, 1.21) | <0.001 |  | 1.03 (1.01, 1.04) | <0.001 | 1.15 (1.11 ,1.18) | <0.001 |
| Cardiovascular disease |  | 1.13 (1.04, 1.22) | 0.003 | 1.31 (1.09, 1.58) | 0.004 |  | 1.08 (1.02, 1.13) | 0.006 | 1.35 (1.18, 1.54) | <0.001 |
| Hypertension ¶¶ |  | 1.74 (1.53, 1.98) | <0.001 | 2.09 (1.52, 2.88) | <0.001 |  | 1.77 (1.64, 1.92) | <0.001 | 2.02 (1.65, 2.48) | <0.001 |
| Lipid-lowering medication |  | 0.93 (0.84, 1.02) | 0.116 | 0.81 (0.65, 1.01) | 0.056 |  | 1.03 (0.97, 1.09) | 0.40 | 0.90 (0.77, 1.05) | 0.16 |

Abbreviation: OR, odds ratio; 95%CI, 95% confidence interval; HDB, Housing and Development Board; HbA_1c_: haemoglobin A_1c_; LDL, low-density lipoprotein; HDL, high-density lipoprotein; BP, blood pressure
 All variables were baseline measurements in 2013. Class 1 was the reference group
† Adjusted for baseline age, gender, and albuminuria at baseline. Single imputation was done for missing values of baseline albuminuria using next observation carried backward method.
‡ BMI at baseline was imputed using the next available follow-up value for the same individual from 2014 to 2019
§ Systolic BP>140 mmHg or Diastolic BP>90 mmHg
¶ Urine albumin creatinine ratio>30 mg/g or abnormal dipstick test
¶¶ Defined as use of any antihypertensive medication

Table S7. Association of baseline characteristics with covariate-adjusted † estimated glomerular filtration rate (eGFR) trajectories based on multiply imputed data‡ (n=62080)

| Variables |  | Class 2 | |  | Class 3 | |  |
| --- | --- | --- | --- | --- | --- | --- | --- |
|  |  | Adjusted OR  (95% CI) | P value |  | Adjusted OR  (95% CI) | P value |  |
| Age≥65 y (vs 18 to 65 y) |  | 1.02 (0.98, 1.07) | 0.37 |  | 0.81 (0.72, 0.91) | <0.001 |  |
| Gender, women |  | 1.28 (1.22,1.33) | <0.001 |  | 1.54 (1.38,1.71) | <0.001 |  |
| Ethnicity |  |  |  |  |  |  |  |
| Chinese |  | 1.00 |  |  | 1.00 |  |  |
| Indian |  | 0.86 (0.81, 0.93) | <0.001 |  | 0.88 (0.75, 1.03) | 0.11 |  |
| Malay |  | 1.34 (1.27,1.42) | <0.001 |  | 1.62 (1.43, 1.83) | <0.001 |  |
| Others |  | 1.09 (0.99, 1.21) | 0.076 |  | 1.42 (1.15, 1.75) | 0.001 |  |
| Housing type |  |  |  |  |  |  |  |
| Condo or landed house |  | 1.00 |  |  | 1.00 |  |  |
| 1 to 2 rooms HDB |  | 1.26 (1.14,1.39) | <0.001 |  | 1.51 (1.19, 1.92) | <0.001 |  |
| 3 to 5 rooms HDB |  | 1.16 (1.08, 1.24) | <0.001 |  | 1.30 (1.07, 1.57) | 0.008 |  |
| Current smoking (vs. not) |  | 1.04 (0.96,1.12) | 0.38 |  | 1.38 (1.18,1.63) | <0.001 |  |
| HbA1C |  |  |  |  |  |  |  |
| <7.0% |  | 1.00 |  |  | 1.00 |  |  |
| ≥7 and ≤9% |  | 1.27 (1.22, 1.33) | <0.001 |  | 1.43 (1.28, 1.61) | <0.001 |  |
| >9.0% |  | 1.76 (1.65, 1.89) | <0.001 |  | 4.66 (4.06, 5.34) | <0.001 |  |
| LDL cholesterol, per 10 mg/dl increase |  | 0.97 (0.96, 0.98) | <0.001 |  | 1.00 (0.98, 1.02) | 0.86 |  |
| HDL cholesterol, per 10 mg/dl increase |  | 0.99 (0.97, 1.00) | 0.15 |  | 0.94 (0.90, 0.98) | 0.007 |  |
| Ln (Triglyceride cholesterol) |  | 1.18 (1.12,1.24) | <0.001 |  | 1.30 (1.15,1.46) | <0.001 |  |
| Uncontrolled BP§ |  | 1.33 (1.27,1.39) | <0.001 |  | 1.81 (1.62, 2.01) | <0.001 |  |
| Albuminuria ¶ |  | 1.40 (1.34,1.45) | <0.001 |  | 2.32 (2.09, 2.57) | <0.001 |  |
| Baseline eGFR, per 10 ml/min/1.73 m^2^ increase |  | 1.13 (1.10, 1.16) | <0.001 |  | 1.02 (1.01, 1.03) | <0.001 |  |
| Cardiovascular disease |  | 1.07 (1.02, 1.12) | 0.007 |  | 1.38 (1.23, 1.54) | <0.001 |  |
| Hypertension ¶¶ |  | 1.78 (1.66, 1.91) | <0.001 |  | 1.78 (1.51, 2.09) | <0.001 |  |
| Lipid-lowering medication |  | 1.02 (0.96, 1.08) | 0.51 |  | 0.93 (0.82, 1.05) | 0.24 |  |

Abbreviation: OR, odds ratio; 95%CI, 95% confidence interval; HDB, Housing and Development Board; HbA_1c_: haemoglobin A_1c_; LDL, low-density lipoprotein; HDL, high-density lipoprotein; BP, blood pressure
 All variables were baseline measurements in 2013. Class 1 was the reference group
† Adjusted for baseline age, gender, and albuminuria at baseline. Single imputation was done for missing values of baseline albuminuria using next observation carried backward method.
‡ 10 imputed datasets by the method of multivariate imputation by chained equations (MICE)
§Systolic BP>140 mmHg or Diastolic BP>90 mmHg
¶ Urine albumin creatinine ratio>30 mg/g or abnormal dipstick test
¶¶Defined as use of any antihypertensive medication

Table S8 Adjusted † association of covariate-adjusted ‡ eGFR trajectory group with ESKD from 2013 to 2019 based on multiply-imputed data§.

| Class | Adjusted HR (95% CI) | P value |  |
| --- | --- | --- | --- |
|  |  |  |  |
| 1 | 1.00 |  |  |
| 2 | 5.26 (4.38, 6.31) | <0.001 |  |
| 3 | 67.68 (50.05, 91.52) | <0.001 |  |

Abbreviation: eGFR, estimated glomerular filtration rate; ESKD, end-stage kidney disease; HR, hazard ratio; 95% CI, 95% confidence interval;
† Adjusted for baseline characteristics including age, gender, ethnicity, housing type, smoking, haemoglobin A_1c_, low-density lipoprotein cholesterol , high-density lipoprotein cholesterol , triglyceride, blood pressure control, albuminuria, cardiovascular disease, hypertension (i.e., antihypertensive medication use), lipid-lowering medication use, and eGFR

‡ Adjusted for age, gender, and albuminuria at baseline. Single imputation was done for missing values of baseline albuminuria using next observation carried backward method.
§ 10 imputed datasets by the method of multivariate imputation by chained equations (MICE)

Figure S1 GFR trajectories and 95% confidence intervals defined by trajectory modelling adjusted for age, gender, and albuminuria at baseline (n=62080). Single imputation was done using next observation carried backward method for missing values of baseline albuminuria. Grey band denotes 95% confidence intervals. The trajectory plot was derived from a 3-class model for a hypothetical 65-year-old woman with no albuminuria at baseline

Trajectory class 1 (Stable initially then gradual decline)
Trajectory class 2 (Progressive decline)
Trajectory class 3 (Rapid decline)


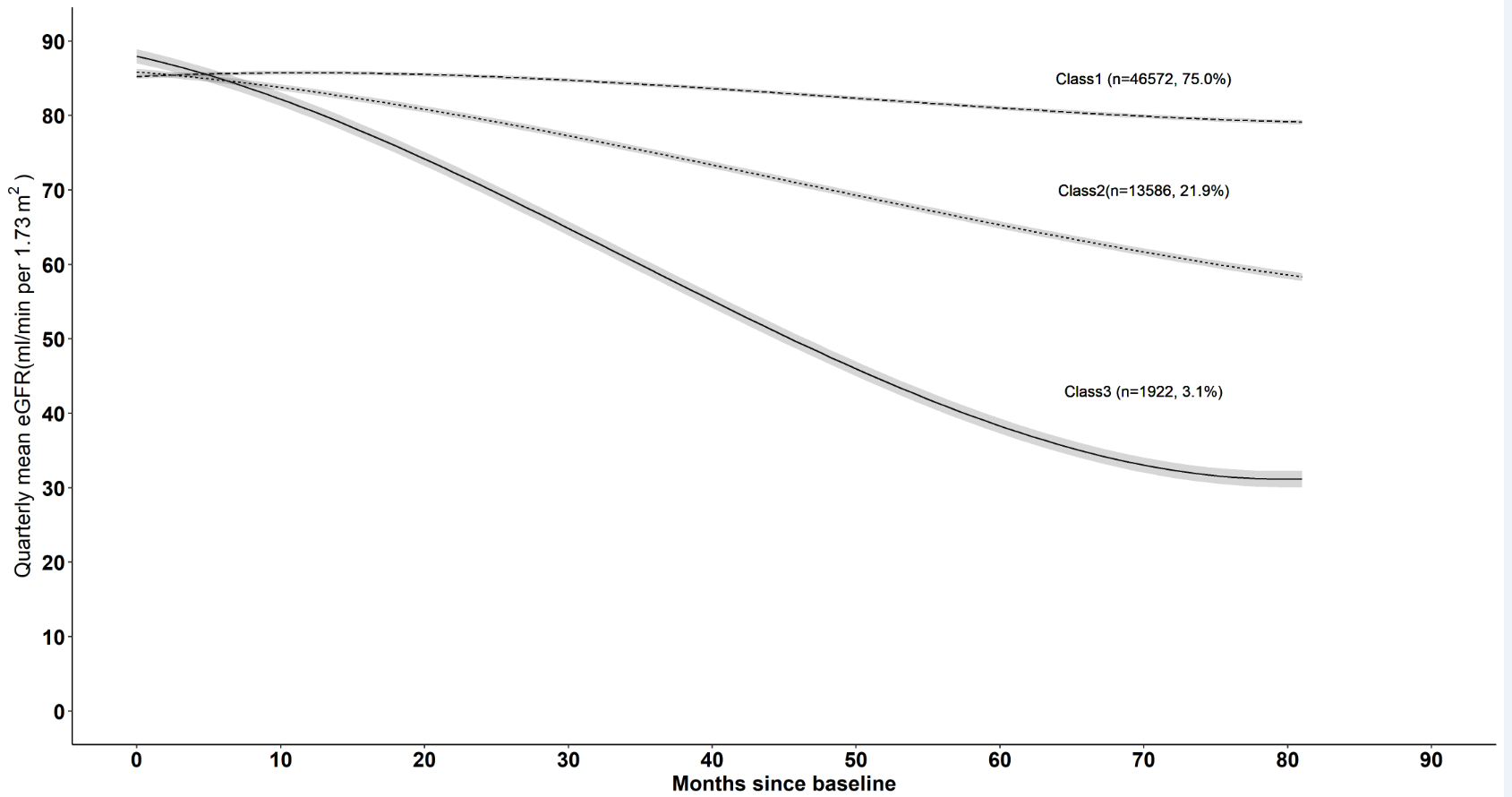


Figure S2 Estimated means with individual trajectories based on the covariate-adjusted model with 3 classes. Covariates adjusted for were age, gender, and albuminuria at baseline. Missing albuminuria was imputed via next observation carried backward method.

Figure S3 GFR trajectories and 95% confidence intervals defined by unadjusted trajectory modelling (n=76045). Grey band denotes 95% confidence intervals

Trajectory class 1 (Stable initially then gradual decline)
Trajectory class 2 (Progressive decline)
Trajectory class 3 (Rapid decline)


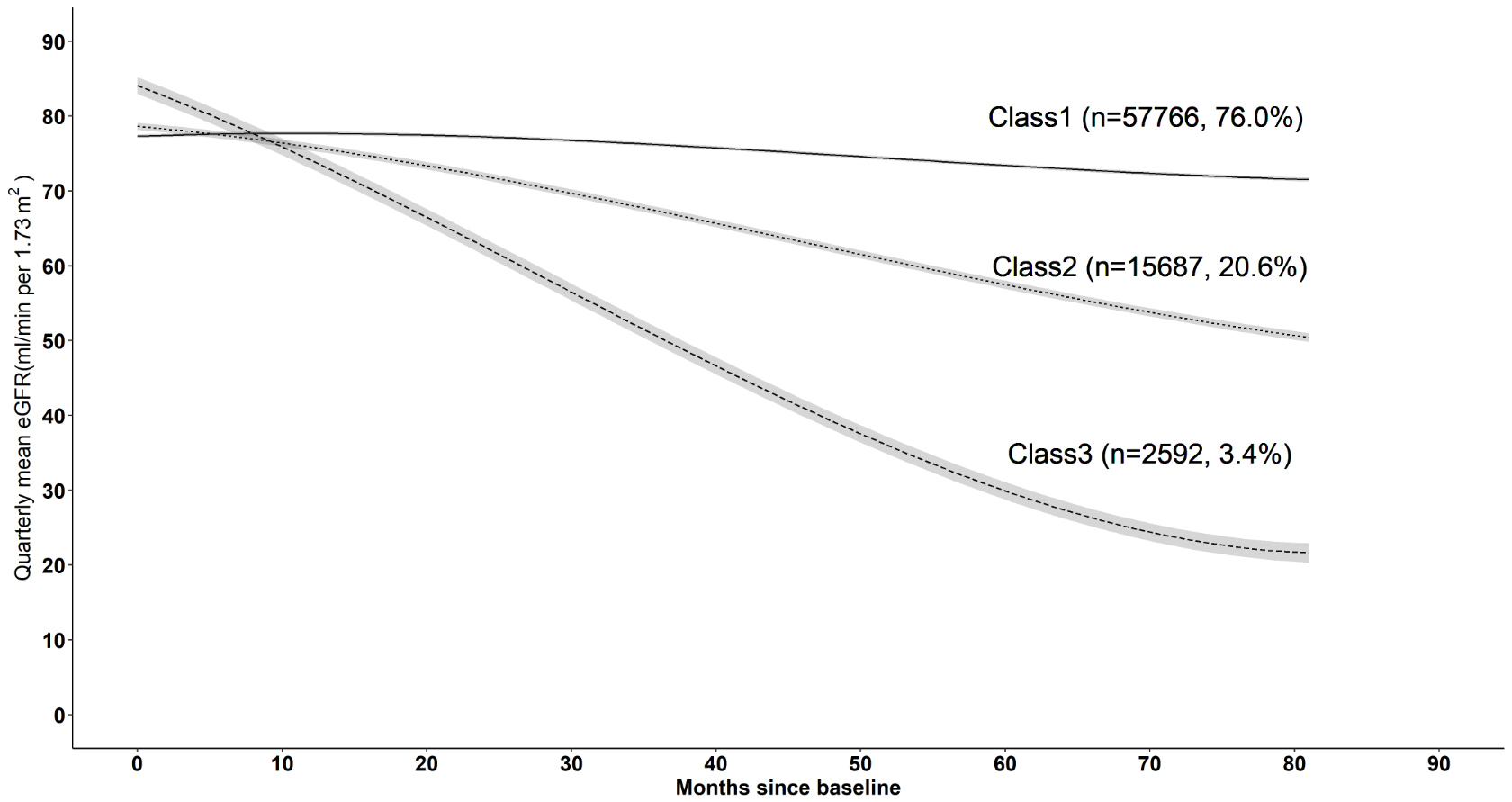


Figure S4 GFR trajectories and 95% confidence intervals defined by trajectory modeling adjusted for age, gender, and albuminuria at baseline (year 2013) (n=76045). Single imputation was done using predictive mean matching method for all missing values of baseline albuminuria. Grey band denotes 95% confidence intervals. The trajectory plot was derived from a 3-class model for a hypothetical 65-year-old man with no albuminuria at baseline

Trajectory class 1 (Stable initially then gradual decline)
Trajectory class 2 (Progressive decline):
Trajectory class 3 (Rapid decline):


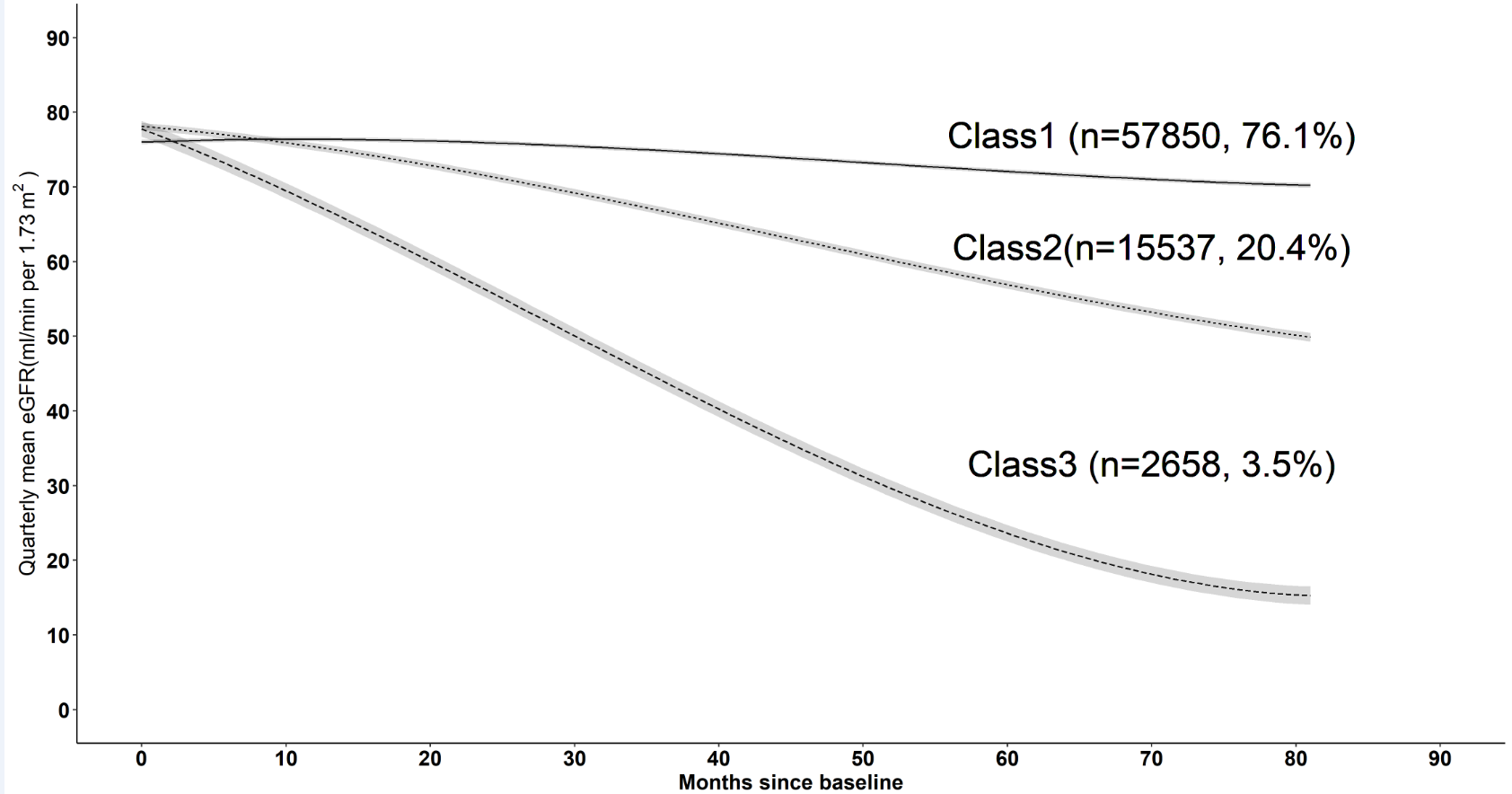

Supplement: Supplementary file 1 — Data S1. Supporting Information. [file JDB-16-e13523-s001.doc]
